# Predictive value of highly sensitive basal versus stimulated thyroglobulin measurement in long-term follow-up of thyroid cancer

**DOI:** 10.1530/EC-22-0312

**Published:** 2023-01-24

**Authors:** Kim Magaly Pabst, Robert Seifert, Nader Hirmas, Martina Broecker-Preuss, Manuel Weber, Wolfgang Peter Fendler, Timo Bartel, Sarah Theurer, Ken Herrmann, Rainer Görges

**Affiliations:** 1 Department of Nuclear Medicine, West German Cancer Center, University Hospital Essen, Essen, Germany; 2 German Cancer Consortium (DKTK), Partner site University Hospital Essen, Essen, Germany; 3 Department of Nuclear Medicine, University Hospital Münster, Münster, Germany; 4 Department of Medicine, Ruhr-University Bochum, University Hospital, Knappschaftskrankenhaus Bochum, Bochum, Germany; 5 Institute of Pathology, University Hospital Essen, University Duisburg-Essen, Essen, Germany

**Keywords:** thyroid cancer, stimulated thyroglobulin, highly sensitive thyroglobulin measurement, unstimulated thyroglobulin, recurrence-free survival

## Abstract

**Objective:**

Recurrence of differentiated thyroid cancer (DTC) is associated with reduced quality of life, and therefore, early identification of patients at risk is urgently needed.Here we investigated the predictive power of various cut-off values of single stimulated thyroglobulin (s-Tg) and single highly sensitive measured, unstimulated thyroglobulin (u-hsTg) measurements close to the end of primary therapy for recurrence-free survival (RFS) in long-term follow-up (>10 years) of patients with DTC.

**Methods:**

In DTC patients with adjuvant radioiodine therapy, we assessed retrospectively u-hsTg (6 ± 3 months before s-Tg measurement) and s-Tg measurements (≤24 months after last radioiodine therapy). Positive predictive (PPV)/negative predictive values (NPV) of various cut-off values (s-Tg: 0.5/1.0 ng/mL; u-hsTg: 0.09/0.2 ng/mL) for patient outcomes as well as additional factors associated with disease development were analyzed.

**Results:**

In total, 175 patients were retrospectively reviewed (tumor recurrence: *n* = 14/complete remission: *n* = 161). Examined cut-off values for s-Tg and u-hsTg showed significant predictive power for RFS (log-rank: all *P* < 0.001). NPV/PPV for s-Tg were 98.6%/36.4%, respectively (0.5 ng/mL cut-off) and 96.7%/42.9%, respectively (1.0 ng/mL cut-off); those for u-hsTg were 97.3%/35.7%, respectively (0.09 ng/mL cut-off) and 95.2%/85.7%, respectively (0.2 ng/mL cut-off). U-hsTg (*P* < 0.001) and patient age (*P* < 0.05) were significantly associated with tumor recurrence. One-third of patients with tumor recurrence in the course initially showed undetectable u-hsTg after completion of primary therapy.

**Conclusion:**

With >10 years of follow-up, both s-Tg and u-hsTg have a comparably high predictive power for RFS, while only u-hsTg was significantly associated with a recurrence event**.**Serial u-hsTg measurements seem warranted since patients with tumor recurrence during follow-up may have an undetectable tumor marker at baseline.

## Key points

- At a follow-up of >10 years, a single stimulated Tg measurement (maximum 24 months after last radioiodine therapy) seems to have a high predictive power for RFS.
- At a follow-up >10 years, a single highly sensitive measured, unstimulated Tg measurement (6 ± 3 months before s-Tg measurement) seems to have also a high predictive power for RFS.
- In head-to-head comparison, only u-hsTg measurements were significantly associated with a recurrence event.
- One-third of patients with tumor recurrence had undetectable u-hsTg at u-hsTg1, indicating that periodic u-hsTg measurements still remain reasonable.
- A statement about the occurrence of tumor recurrence is not possible for a follow-up >10 years.

## Introduction

Current guidelines for differentiated thyroid carcinoma (DTC) (1, 2) assign a crucial diagnostic importance to stimulated thyroglobulin (s-Tg) measurement 6–12 months after completion of primary therapy to predict a recurrence-free follow-up than in previous guideline versions.

This is mainly based on publications by Kloos *et al.* (3) and Castagna *et al.* (4). While Kloos *et al.* set an s-Tg value <0.5 ng/mL as a cut-off for a tumor-free follow-up of 3–5 years, Castagna *et al.* use a cut-off value of <1.0 ng/mL. However, the predictive power in the long-term course (>5 years) also seems to be decisive, since the cumulative recurrence rate in the overall course of DTC is reported to be up to 30%, and some of these recurrences occur years to decades after primary therapy has been completed (5).

In 2019, Giovanella *et al.* (6) published a meta-analysis on the predictive power of highly sensitive measured, unstimulated thyroglobulin (u-hsTg) under ongoing l-thyroxine treatment for the detection of recurrence, as an alternative to s-Tg measurement. With a negative predictive value (NPV) of 99.4%, u-hsTg at a cut-off of <0.1 ng/mL could also be classified as a strong prognostic predictor. However, the follow-up time was likewise limited to a maximum of 64 months (6).

Primary endpoint of this study was to determine the predictive power for recurrence-free survival (RFS) of the different cut-off values of s-Tg (≤24 months after completion of primary therapy) and u-hsTg (6 ± 3 months before s-Tg measurement) for a longer follow-up period (up to a maximum of 16 years) compared to the aforementioned studies. As secondary endpoint, we investigated the extent to which u-hsTg is a suitable predictor compared to s-Tg measurement at the time point of radioiodine diagnostics. Furthermore, demographic factors likely associated with tumor recurrence (such as age, sex, and others) were investigated.

## Materials and methods

### Patient population

The institutional database of the Department of Nuclear Medicine at the University Hospital Essen was screened for patients who underwent at least one radioiodine therapy due to DTC between May 2005 and May 2011. The tumor marker thyroglobulin (Tg) was retrospectively evaluated at different time points after completion of radioiodine ablation due to DTC. Patients were included if u-hsTg measurements were available after completion of radioiodine therapy (6 ± 3 months before s-Tg measurement as part of radioiodine diagnostic procedure), as well as an s-Tg measurement (maximum 24 months after last radioiodine ablation) and at least annual follow-ups (including u-hsTg and TSH measurements, as well as ultrasonography). The study was approved by the local ethics committee at the University Hospital Essen (15-6702-BO).

Since the initial tumor diagnosis was made many years ago and the TNM classification has changed several times in the meantime (fifth edition (1997), sixth edition (2002), seventh edition (2009), and eighth edition (2016)), the tumor stage of each patient was re-classified using a TNM classification by applying the eighth edition (2016) of the AJCC/UICC.

### Study design and follow-up

Retrospectively, the tumor marker thyroglobulin (including Tg antibodies (TgAb) measurement and/or recovery test for authentication of the measured Tg values), as well as TSH level, were evaluated at different time points after completion of radioiodine therapy due to DTC. In all patients, Tg and TSH measurements were always performed on the same day.

Time-point u-hsTg1 included the evaluation of an u-hsTg measurement in the context of a follow-up examination under l-thyroxine medication (6 ± 3 months before s-Tg measurement; first u-hsTg measurement after completion of radioiodine therapy). The corresponding TSH value for u-hsTg measurement was chosen to be <0.5 mU/L, so that patients had a decreased or low-normal TSH range, and the influence of TSH level on Tg was negligible (1, 2, 7, 8).

In accordance with current guidelines of the American Thyroid Association (ATA) and the British Thyroid Association (BTA) (1, 2), an endogenous (previous l-thyroxine withdrawal for 4 weeks) s-Tg measurement was performed at time point s-Tg2 (maximum 24 months after last radioiodine therapy), requesting a TSH value ≥30 mU/L as part of a radioiodine diagnostic procedure (2).

All patients were followed up on average 1–2 times annually: TSH and u-hsTg measurements (along with TgAb and/or recovery testing), as well as ultrasonography examinations, were performed. The primary endpoint of the study was to evaluate the predictive power of different s-Tg and u-hsTg cut-off values for the prediction of RFS for a period >5 years.

Complete remission was defined as an absence of morphologic/biochemical tumor evidence for at least 5 years with an u-hsTg < 0.2 ng/mL at last follow-up (u-hsTg3), in accordance with the ATA response criteria (1). Structural disease (detected at least by [18F]F-FDG PET/CT scan or histological confirmation) and biochemical recurrence (biochemical incomplete response (for further information see Table 1) with continuously increasing Tg over time) were considered as tumor recurrence in this analysis. In patients with tumor recurrence, the last u-hsTg before diagnosis of recurrence was also recorded (u-hsTg3) in Supplementary Table 2 (see section on supplementary materials given at the end of this article).

**Table 1:** Patient outcomes according to the American Thyroid Association response criteria (1).

Category	Definitions	*n* (%)	Sex (f:m)	u-hsTg3 (last follow-up/before tumor recurrence); ng/mL (±s.d.)
Excellent response	Negative imaging **and**either suppressed Tg < 0.2 ng/mL or TSH-stimulated Tg < 1 ng/mL	161 (88.5)	2.4:1	0.14 ± 0.04
Biochemical incomplete response	Negative imaging **and** suppressed Tg ≥ 1 ng/mL **or** stimulated Tg ≥ 10 ng/mL **or** rising anti-Tg antibody level	1 (0.5)	1:0	39
Structural incomplete response	Structural or functional evidence of disease with any Tg level with or without anti-Tg antibodies	13 (7.1)	1:1.6	158.5 ± 526.9
Indeterminate response^a^	Nonspecific findings on imaging studies, faint uptake in thyroid bed on RAI scanning, non-stimulated Tg detectable but <1 ng/mL, stimulated Tg detectable but <10 ng/mL **or**anti-Tg antibodies stable or decline in the absence of structural or functional disease	7 (3.8)	1:1.3	0.26 ± 0.09

^a^Indeterminate response was excluded in further analysis to keep the probability of tumor recurrence low after completion of the follow-up period.

RAI, radioactive iodine; SD, standard deviation.

Consequently, patients with a complete remission were followed retrospectively for at least 5 years but mostly longer (median 11 years). Patients with tumor recurrence were followed up at least until the tumor disease recurred but mostly until last follow-up at the University Hospital Essen. Patients with an indeterminate response were not included in the evaluation, in order to keep the probability of tumor recurrence low after completion of the follow-up period. An indeterminate response is defined as u-hsTg > limit of quantitiation (LoQ) but <1.0 ng/mL and/or s-Tg >LoQ but <10 ng/mL in the absence of structural/functional disease (1). The likelihood of structural disease occurring in further follow-ups is 15–20% (9, 10).

TSH, free T3 (fT3), and free T4 (fT4) were measured using the ADVIA Centaur XPT Immunoassay system, and TgAb levels were measured using the Immulite 2000XPi Immunoassay system (both Siemens Healthineers) at the different time points mentioned previously.

### Thyroglobulin assays

For quantitative thyroglobulin measurement, a highly sensitive enzyme-linked immunosorbent assay (ELISA) with an LoQ in our laboratory of 0.09 ng/mL (Medizym® Tg Rem ELISA; Medipan, Blankenfelde-Mahlow, Germany) was used. As described in the current ATA guidelines (1, 8), a suppressed Tg <0.2 ng/mL was chosen as a cut-off value as part of an excellent response to tumor treatment. For Tg values above 3 ng/mL, measurement of the tumor marker was performed by IRMA with an LoQ < 0.5 ng/mL (SELco® Medizym Tg IRMA; Medipan, Dahlewitz, Germany). Authentication of Tg values was done by performing TgAb measurement (TgAb <functional assay sensitivity) and/or recovery test. The defined range for undisturbed recovery was 60–140% in TgREM assay (low-dose recovery test) and 70–130% in SELco Medizym Tg assay (conventional recovery test). We have previously published data on both assays in detail (11, 12).

### Statistical analysis

Statistical analysis was performed using SPSS version 27.0 (IBM). For continuous data, median, mean, and range were reported, while categorical variables were described using frequencies. Sensitivity, specificity, NPV, and positive predictive value (PPV) were calculated for the analysis of the predictive power of the different s-Tg and u-hsTg cut-off values (s-Tg: 0.5 ng/mL and 1.0 ng/mL; u-hsTg: 0.2 ng/mL and 0.09 ng/mL) on RFS. Kaplan–Meier curves were used for graphical representation. Statistical significance was evaluated by log-rank test. A *P*-value of <0.05 was considered statistically significant. In addition, multivariate Cox regression was performed to examine the association of various factors with tumor recurrence. Tg levels clinically reported less than 0.09 ng/mL are considered as 0.045 ng/mL for statistical purposes.

## Results

### Patients and follow-up

In total, 182 patients with DTC were examined. Seven patients (female *n* = 3; male *n* = 4; 3.8%) were excluded because of an indeterminate therapy response.

Overall, 120 female (69%) and 55 male (31%) patients were included in this analysis. *n* = 14 patients (8%) showed a tumor recurrence, of whom 13 patients had structural disease on ultrasonography and/or PET/CT (ultrasonography: *n* = 3, [^18^F]F-FDG-PET/CT: *n* = 9 patients, [^18^F]F-FDG-PET/CT and Iod-124 PET/CT: *n* = 3 patients), and *n* = 7 had histological confirmation of disease. One patient showed biochemical recurrence with a continuous u-hsTg increase over time to a final value of 39 ng/mL. Overall, 19.4% (7/36) of patients with FTC and 5.1% (7/137) of patients with PTC showed tumor recurrence. Tumor recurrences were subdivided into ten early recurrences (≤5 years) and four late recurrences (>5 years). The average follow-up time for patients with tumor recurrence was 10.5 years (median: 11 years; range: 4–16 years). Further information is included in Supplementary Tables 1 and 2. In total, 161 patients (92%) had a complete remission with an average follow-up period of 10.6 years (median: 11 years; range: 5–16 years). A summary of patient outcomes in terms of the ATA response criteria is shown in Table 1.

All patients had undergone at least one radioiodine therapy with a median activity of 6 GBq (mean: 6.1 GBq; range: 3–16 GBq). At time u-hsTg1 (median: 185 days after completed radioiodine therapy; mean: 197.1 days; range: 77–587 days), u-hsTg averaged 0.1 ng/mL (median: 0.09 ng/mL; range: <0.09–0.64 ng/mL) and TSH 0.07 mU/L (median: 0.03 mU/L; range: 0.01–0.48 mU/L).

At time s-Tg2 (median: 366 days after completed radioiodine therapy; mean: 12 months; range: 5–24 months), assuming TSH > 30 mU/L (2), s-Tg averaged 0.84 ng/mL (median: 0.14 ng/mL; range: <0.09–44 ng/mL).

Patients with complete remission all presented an u-hsTg < 0.2 ng/mL at timepoint u-hsTg3 (median: <0.09 ng/mL, range: <0.09–0.19 ng/mL). When tumor recurrence occurred, the median u-hsTg before detection of recurrence (u-hsTg3) was 2.35 ng/mL (range: 0.38–1911 ng/mL).

### Tumor characteristics and stage

In total, 175 patients with DTC were examined, of whom 137 patients (78%) had papillary and 36 patients (21%) had follicular thyroid carcinoma. Two patients (1%) had concomitant follicular and papillary thyroid carcinoma.

Two patients had distant metastases at initial diagnosis (distant lymph node metastasis and pulmonary metastases); however, in both, the tumor lesions detected in posttherapy scan of the first radioiodine therapy could no longer be detected in the posttherapy scan of the second radioiodine therapy including [^18^F]F-FDG PET/CT (to exclude dedifferentiated tumor parts). Furthermore, these patients showed an u-hsTg below LoQ at u-hsTg1.

Around 174 patients (99.4%) could be assigned to UICC stage I and II in total (according to the eighth edition of TNM classification (2016); stage I: 148 patients (85.1%), stage II: 26 patients (14.9%)). Higher-grade stages (III–IVb) occurred in one (0.6%) patient (stage III: 1 patient (100%)). For more details, see Tables 2 and 3.

**Table 2:** Overview of tumor histology and TNM status (*n*  = 175).

Tumor histology and TNM/UICC status^a^		*n* (%)
Histology	Papillary	137 (78.3)
	Follicular	36 (20.6)
	Papillary and follicular	2 (1.1)
TNM	T1a	30 (17.1)
	T1b	55 (31.4)
	T2	46 (26.3)
	T3a	17 (9.7)
	T3b	2 (1.1)
	T3^b^	21 (36.8)
	T4a	3 (1.7)
	T4b	1 (0.6)
	N0	145 (83.7)
	N1^c^	4 (2.3)
	N1a	11 (6.3)
	N1b	15 (8.6)
	M0	173 (98.9)
	M1	2 (1.1)
UICC	I	148 (84.6)
	II	26 (14.9)
	III	1 (0.6)
	IVa	–
	IVb	–

^a^TNM/UICC staging is based on the 8th edition of the TNM classification (2016) at first radioiodine therapy;

^b^T3: Patients whose primary tumor could not be retrospectively assigned to any category (T3a/T3b); ^c^N1: Patients whose lymph node metastases could not be retrospectively assigned to any category (N1a/N1b).

**Table 3:** Comparison of different parameters in patients with complete remission and patients with tumor recurrence.

Characteristics	Complete remission	Tumor recurrence
*n* (%)	161 (92)	14 (8)
Age (±s.d.)	48.4 ± 14.7	56.9 ± 15
Sex (f:m)	2.4:1	1:1.3
Histology		
PTC (%)	130 (80.7)	7 (50)
FTC (%)	29 (18.0)	7 (50)
PTC and FTC (%)	2 (1.2)	–
UICC (eighth edition 2016) stage at initial RIT		
1 (%)	140 (87.0)	8 (57.1)
2 (%)	21 (13.0)	5 (35.7)
3 (%)	–	1 (7.1)
4 (%)	–	–
u-hsTg1 (ng/mL; range)	<0.09 (<0.09–0.48)	0.25 (<0.09 –0.64)
s-Tg2 (ng/mL; range)	0.46 (<0.09–18)	4.97 (<0.09–44)
GBq (in total; range)	6.5 (3–9)	5.6 (2–7)

FTC, follicular thyroid carcinoma; GBq, gigabecquerel; PTC, papillary thyroid carcinoma; RIT, radioiodine therapy; s-Tg, stimulated Tg; u-hsTg, highly sensitive measured, unstimulated thyroglobulin; u-hsTg1, u-hsTg measured 6 ± 3 months before s-Tg; s-Tg2, s-Tg measured after u-hsTg1 (maximum 24 months after last radioiodine therapy).

### Negative/positive predictive values for stimulated Tg-measurement

Cut-off values of 0.5 and 1.0 ng/mL, similar to the ones used by Castagna *et al.* (4) and Kloos *et al.* (3), were chosen for the calculation of NPV and PPV, respectively, of s-Tg for the entire patient cohort.

**Figure 1:**
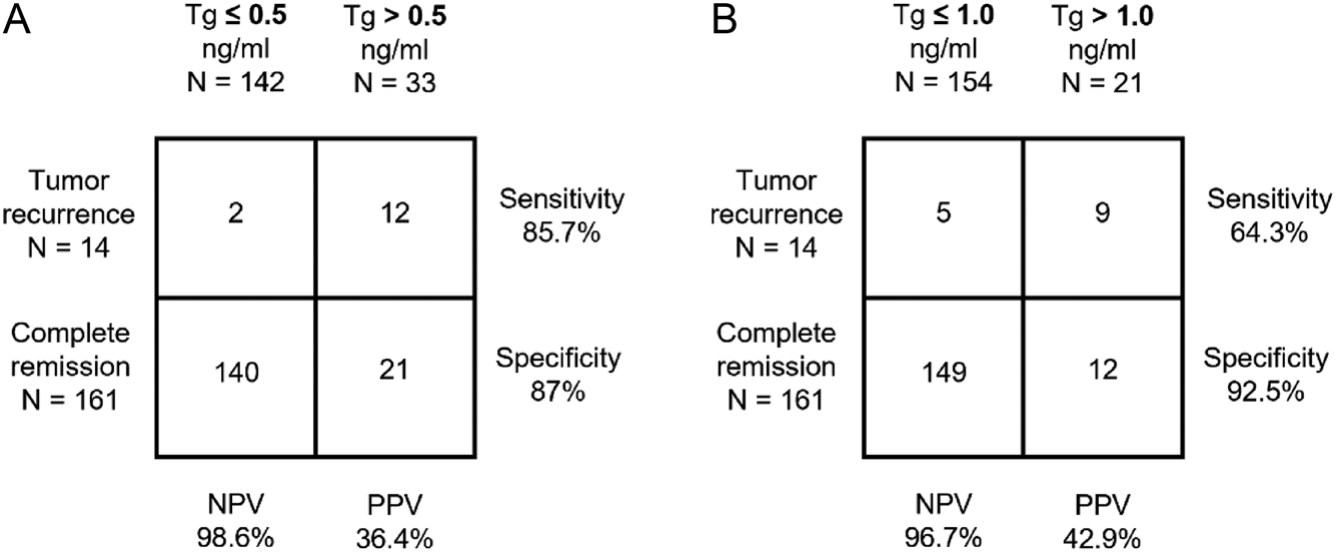
Test accuracy of stimulated thyroglobulin measurement for patient outcomes. Test accuracy of s-Tg cut-off values of (A) 0.5 ng/mL and (B) 1.0 ng/mL (maximum 24 months after last radioiodine therapy) for the entire patient cohort.

The NPV at a Tg cut-off value of ≤0.5 ng/mL was 98.6%, while the PPV was 36.4%. The other investigated cut-off value of ≤1.0 ng/mL yielded an NPV of 96.7% and a PPV of 42.9%. Further information is given in Fig. 1.

**Figure 2:**
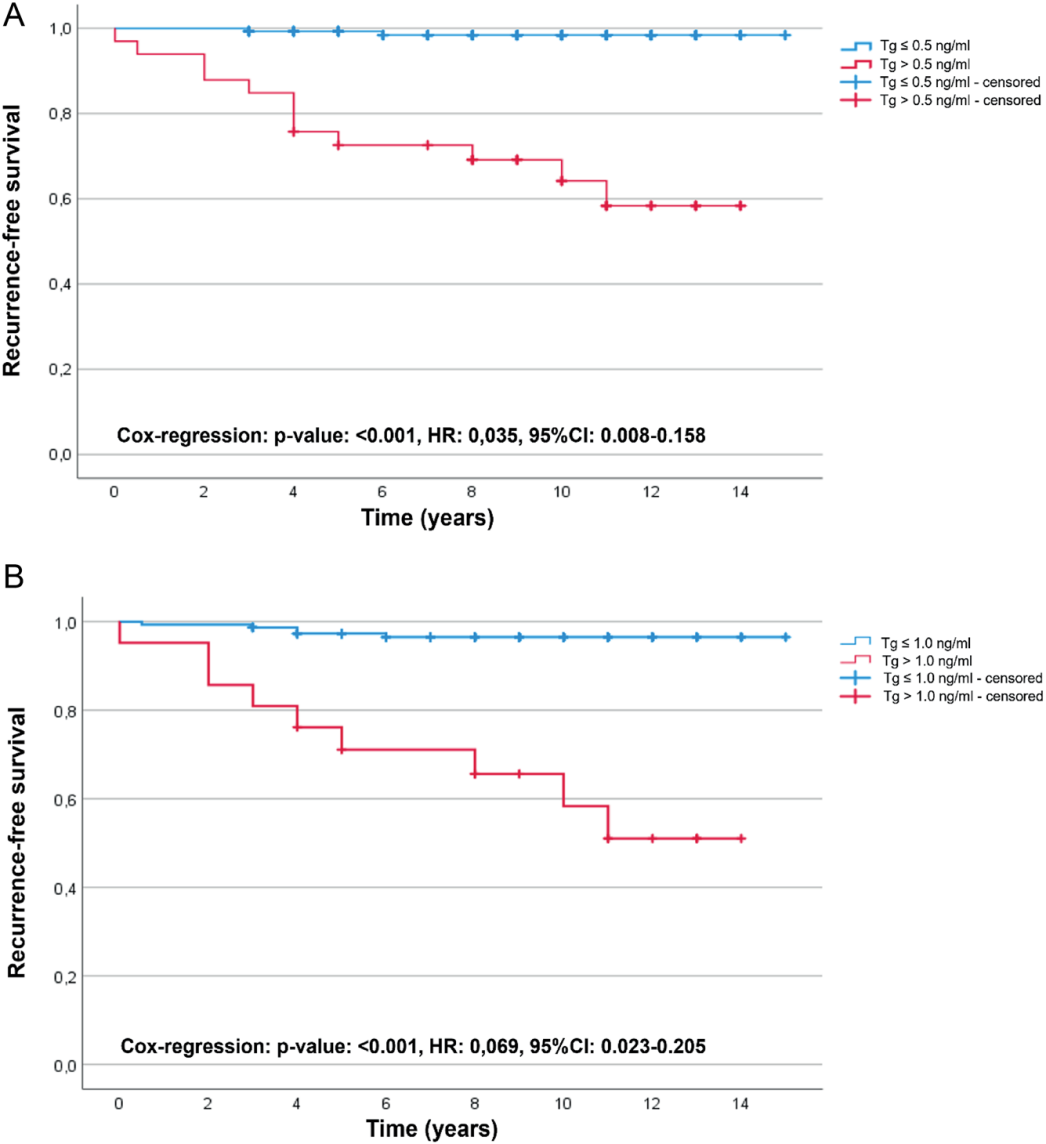
Recurrence-free survival depending on different stimulated thyroglobulin levels. Kaplan–Meier curves showing recurrence-free survival (RFS) of patients with (A) s-Tg (maximum 24 months after last radioiodine therapy) ≤0.5 ng/mL vs >0.5 ng/mL and (B) s-Tg ≤1.0 ng/mL vs >1.0 ng/mL. HR, hazard ratio.

Both cut-off values showed significant predictive power via log-rank test (Tg ≤ 0.5 ng/mL vs Tg > 0.5 ng/mL, and Tg ≤ 1.0 ng/mL vs Tg > 1.0 ng/mL; both *P* < 0.001) for RFS, even at a significantly longer follow-up period (> 10 years). Kaplan–Meier curves are shown in Figs. 2A and B.

### Negative/positive predictive values for unstimulated Tg-measurement

**Figure 3:**
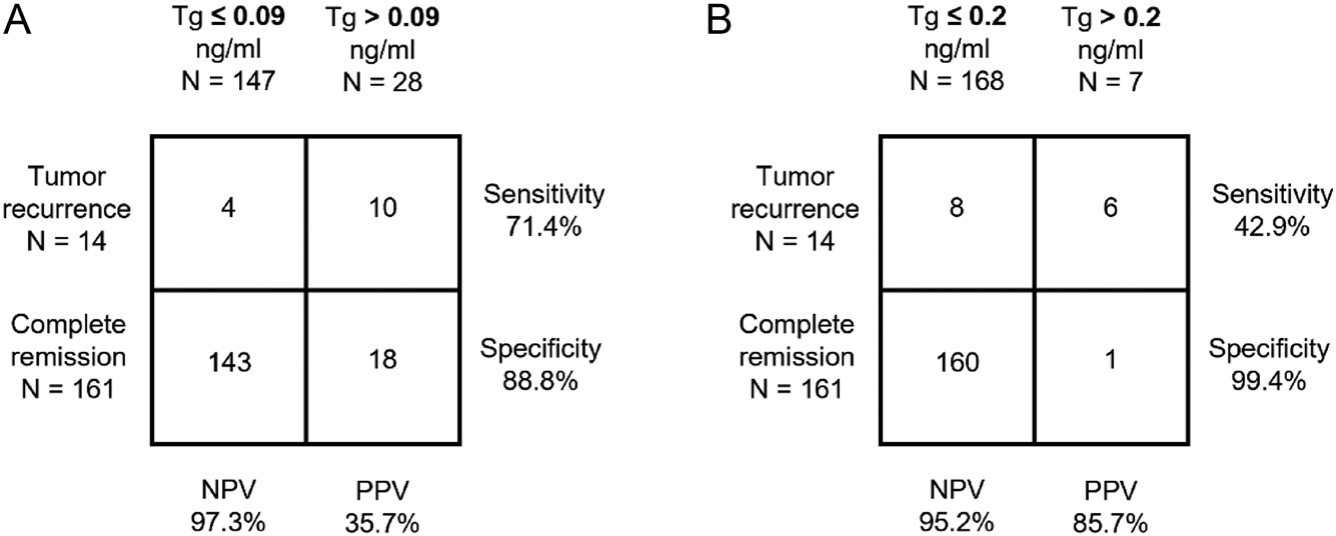
Test accuracy of highly sensitive, unstimulated thyroglobulin measurement for patient outcomes. Test accuracy of u-hsTg cut-off values of (A) 0.09 ng/mL and (B) 0.2 ng/mL (6 ± 3 months before s-Tg2 as part of radioiodine diagnostics) for the entire patient cohort.

The condition for inclusion was, as previously described, a TSH level <0.5 mU/L at u-hsTg1 to minimize the influence of TSH level on tumor marker Tg (1, 2, 7, 8). The mean unstimulated TSH value was 0.07 mU/L (median: 0.03 mU/L; range: 0.01–0.48 mU/L). The selected cut-off value of ≤0.2 ng/mL corresponds to the ‘excellent response’ category of the current ATA guidelines (1) with a 1–4% likelihood of tumor recurrence (3, 9, 13, 14). The NPV for the u-hsTg measurement was 95.2% and the PPV was 85.7% (Fig. 3B).

To accommodate the improvement of functional sensitivity of Tg assays, NPV and PPV were further analyzed with a cut-off value of <0.09 ng/mL – corresponding to the LoQ of the applied, highly sensitive ELISA (Fig. 3A). The results were comparable to those of the s-Tg measurement at a cut-off value of ≤0.5 ng/mL (NPV: 97.3%, PPV: 35.7%).

**Figure 4:**
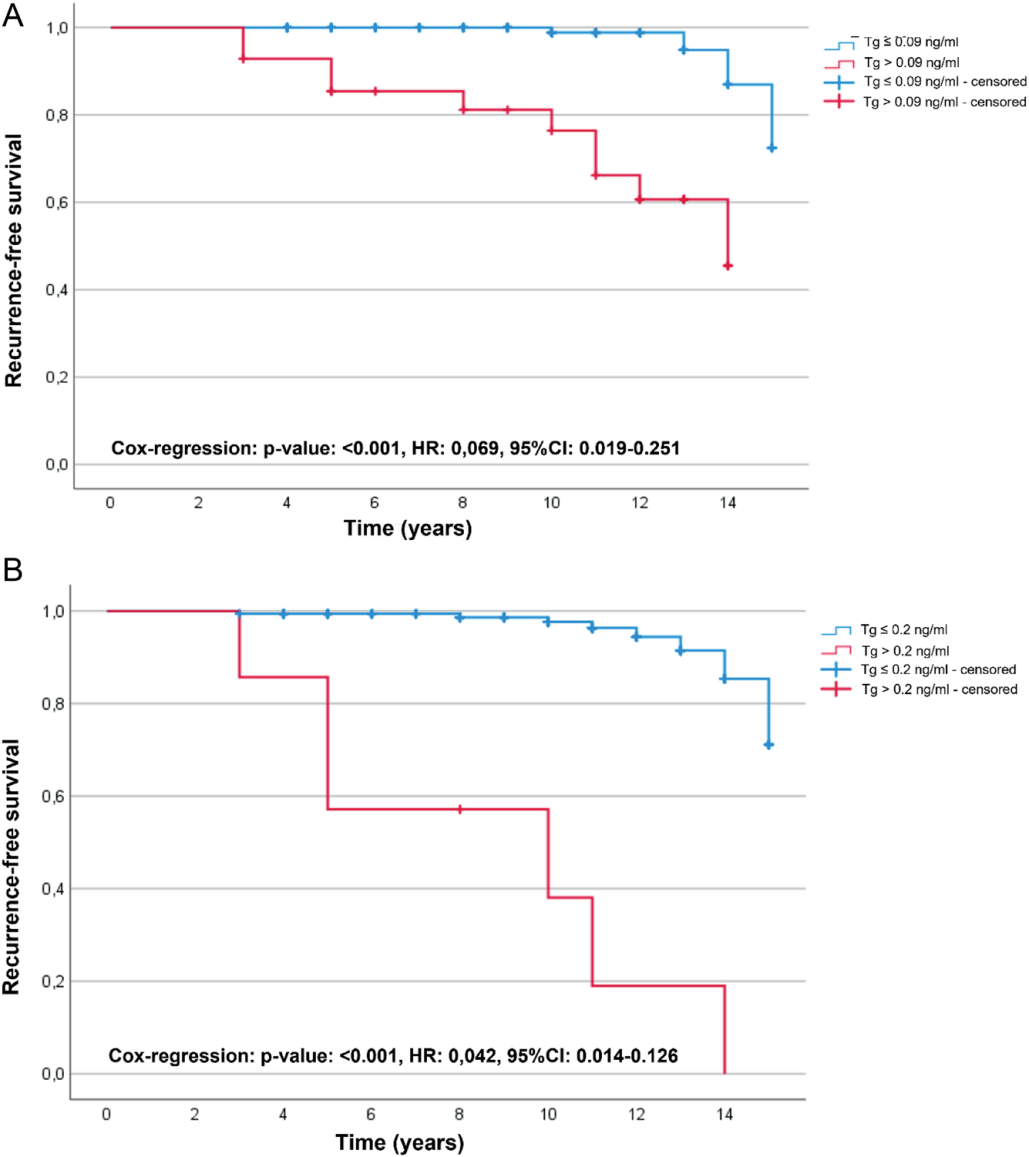
Recurrence-free survival depending on different highly sensitive, unstimulated thyroglobulin levels. Kaplan–Meier curves plotting recurrence-free survival (RFS) of patients with u-hsTg (6 ± 3 months before s-Tg measurement as part of radioiodine diagnostics) ≤0.09 ng/mL vs >0.09 ng/mL (A) and in panel (B), with u-hsTg ≤ 0.2 ng/mL vs >0.2 ng/mL; TSH level <0.5 mU/L. HR, hazard ratio.

Again, the cut-off values showed a clearly significant predictive power (u-hsTg ≤ 0.2 ng/mL vs u-hsTg > 0.2 ng/mL and u-hsTg ≤ 0.09 ng/mL vs u-hsTg > 0.09 ng/mL; both *P* < 0.001) for RFS using the log-rank test, as shown in the Kaplan–Meier curves in Figs. 4A and B.

### Association of various factors with tumor recurrence

To examine the association of various factors with tumor recurrence, including age, sex, and tumor stage (UICC eighth edition), as well as s-Tg, u-hsTg, and treatment activity (GBq), multivariate Cox regression was performed (more details are provided in Table 4). The omnibus test showed a significant result for this parameter constellation (*P* < 0.001).

**Table 4:** Analysis of the association of various factors with tumor recurrence in patients suffering from differentiated thyroid carcinoma^a^.

Covariate	Regression coefficient	*P*-value	Hazard ratio	95% CI of odds ratio	
				Lower limit	Upper limit
Sex	−1.153	0.084	0.316	0.085	1.169
Age	0.118	0.005	1.125	1.037	1.220
Activity (GBq)	0.288	0.057	1.333	0.992	1.792
u-hsTg (ng/mL)	6.006	<0.001	405.823	16.292	10108.961
s-Tg (ng/mL)	0.06	0.078	1.061	0.993	1.134
Tumor stage					
I		0.488			
II	−0.936	0.451	0.382	0.031	4.657
III	−0.254	0.854	0.776	0.052	11.681

^a^Multivariate Cox regression to assess the association of age, sex, tumor stage (UICC eighth edition), treatment activity (GBq), as well as u-hsTg (ng/mL) and s-Tg (ng/mL) with tumor recurrence.

GBq, gigabecquerel; s-Tg, stimulated Tg; u-hsTg, highly sensitive measured, unstimulated thyroglobulin .

In this multivariate analysis, u-hsTg (*P* < 0.001) and age (*P* < 0.05) showed a significant, positive association with patient outcomes regarding tumor recurrence, whereas s-Tg (*P* = 0.078), therapy activity (*P* = 0.057), as well as tumor stage and sex (*P* = 0.084) are not significant parameters in our patient cohort.

Also, in head-to-head comparison, only u-hsTg was significantly associated with tumor recurrence, whereas s-Tg was not significant here (more details are provided in Table 5).

**Table 5:** Head-to-head comparison of the association of single stimulated thyroglobulin and single highly sensitive measured, unstimulated thyroglobulin measurement with the occurrence of tumor recurrence in multivariate Cox-regression.

Covariate	Regression coefficient	*P*-value	Hazard ratio	95% CI of odds ratio	
				Lower limit	Upper limit
u-hsTg (ng/mL)	4.93	<0.001	138.341	12.523	1528.188
s-Tg (ng/mL)	0.04	0.112	1.04	0.991	1.092

s-Tg, stimulated Tg; u-hsTg, highly sensitive measured, unstimulated thyroglobulin.

## Discussion

To the best of our knowledge, the present study is currently unique as it examines and compares the prognostic power of u-hsTg and s-Tg regarding RFS in patients with DTC over a median time of 11 years.

Overall, we confirmed the well-known excellent prognosis for patients with DTC (15). In our cohort, only 14 of 175 patients showed tumor recurrence during follow-up. Worth mentioning here is that tumor recurrence occurred in 19.4% of all patients with FTC and only in 5.1% of patients with PTC. These results are largely consistent with the statements of previous publications (1, 16).

Another important aspect of this study is that the majority (71.4%) of patients with tumor recurrence had early recurrence (≤5 years), however, remaining 28.6% showed late recurrence (>5 years). Mazzaferri *et al.* previously declared that over 30% of patients with DTC develop tumor recurrence over 3 decades, depending on their initial therapy. Two-third of these recurrences occur within the first decade, and the remainder years later (17). This highlights the limitations of the studies (3, 4) on which the current guidelines are based (1, 2): With a follow-up of 3–5 years, these studies could not adequately capture the mentioned recurrence rates.

To bridge this gap, the present retrospective study examined s-Tg cut-off values (0.5 and 1.0 ng/mL) regarding their predictive power for RFS for a significantly longer time interval with a median of 11 years. Here, similar behavior of NPVs is demonstrated for the two s-Tg cut-off values (0.5 and 1.0 ng/mL, TSH 30 mU/L) for a period >10 years.

Whereas Kloos *et al.* presented an NPV of 98.5% for an s-Tg cut-off of 1.0 ng/mL (3), our current study showed a very close NPV for a follow-up >10 years (96.7%). Castagna *et al.* set an s-Tg cut-off value of 0.5 ng/mL with an NPV of 98% (4), while the current data show a similar NPV (98.6%). In conclusion, s-Tg measurement is highly predictive of RFS for a follow-up period of even >10 years. However, this contrasts with a markedly lower PPV in the current study (s-Tg ≤ 0.5 ng/mL: PPV 36.4% vs PPV 80% (3); Tg ≤ 1.0 ng/mL: PPV 42.9%), which can be explained, among others, by the long follow-up period.

More recent studies (6, 8) elucidate an equivalent NPV of u-hsTg measurement for patient outcome compared to s-Tg measurement and therefore, postulate a lack of need for s-Tg measurement in patients with an undetectable u-hsTg (6, 18).

In 2014, Giovanella *et al.* (19) applied an u-hsTg cut-off value of 0.1 ng/mL and demonstrated a comparable NPV to the s-Tg measurements of Kloos *et al.* and Castagna *et al.* (3, 4). A low PPV of the u-hsTg measurement was described as a disadvantage (19). Consequently, in case of a detectable u-hsTg, an s-Tg measurement should still be aimed for (19). On the other hand, Spencer *et al.* (20) showed a correlation between s-Tg and u-hsTg measurements and recommended basal Tg measurement for long-term follow-up without repetitive s-Tg measurements for most DTC patients. Börgershausen *et al.* (15) confirmed the value of u-hsTg as a prognostic marker in long-term follow-up in 2022.

Over time, methods for highly sensitive Tg measurement have evolved. In the meanwhile, LoQ of 0.09 ng/mL is recorded and low-dose recovery test is applied. To adapt to such circumstances, we did not only explore the aforementioned cut-off value of 0.2 ng/mL (according to the ‘excellent response’ category of the current ATA guidelines (1)), but we have also additionally used the LoQ of the utilized highly sensitive ELISA as a cut-off value (0.09 ng/mL).

As expected, according to the previously mentioned publications (19), both u-hsTg cut-off values show high NPVs (u-hsTg: 0.09 ng/mL: 97.3% vs u-hsTg: 0.2 ng/mL: 95.2%). A slightly higher NPV is shown at a cut-off value of 0.09 ng/mL to the disadvantage of the PPV (u-hsTg: 0.09 ng/mL: 35.7% vs u-hsTg: 0.2 ng/mL: 85.7%). The results for a cut-off value of 0.09 ng/mL correspond to those of s-Tg measurement by Castagna *et al.* Hence, the previously mentioned results of Giovanella *et al.* (19) are confirmed and indicate that a standardized s-Tg measurement is redundant.

This seems to be reconfirmed by the multivariate Cox regression performed in our study, since the head-to-head comparison of s-Tg and u-hsTg does not reveal a significant association of s-Tg measurement with a recurrence event in the investigated cohort. However, it should be mentioned that other confounding factors not captured by our retrospective study design might be the cause of missing association between s-Tg and tumor recurrence.

Using multivariate Cox analysis, age was also shown to have a positive association with the occurrence of tumor recurrence during follow-up >10 years in patients with DTC. These results are in line with other publications (21, 22). Additional factors, such as sex, treatment activity, and tumor stage demonstrated no significant association in our cohort.

In summary, for both the selected s-Tg and u-hsTg cut-off values, a high NPV and thus an impressive predictive power of RFS could also be established for a follow-up period of more than 10 years. In line with the aforementioned publications (3, 4, 19), PPVs continue to be insufficient to predict tumor recurrence at follow-up of more than 10 years. A statement about the occurrence of tumor recurrence is not possible with certainty at low PPV for such a long time period. Nevertheless, that a single u-hsTg/s-Tg measurement is not entirely capable of predicting patient outcome is evident when considering any individual with tumor recurrence (Supplementary Table 2). 35.7% (5/14) demonstrated a tumor marker below the detection limit of 0.09 ng/mL/57.1% (8/14) ≤0.2 ng/mL at u-hsTg1, 35.7% (5/14) were below the cut-off of 1.0 ng/mL/14.3% (2/14) ≤ 0.5 ng/mL at s-Tg2.

In accordance with Giovanella *et al.*, periodic (serial) highly sensitive, unstimulated thyroglobulin measurements could compensate for this shortcoming, because tumor recurrence is detected early on due to a continuous rise (8). The usefulness of this approach has already been confirmed by other publications (23, 24, 25).

Our study is limited by the excellent outcome of patients with DTC. Due to a low recurrence rate (*n*  = 14), our results can only be considered exploratory. Our analysis warrants the conduct, urgently needed, of prospective studies with a larger cohort. However, since we are already a tertiary hospital, it will be difficult to obtain monocentric larger cohorts.

Further limitations include the retrospective study design and the biased population, which contained only patients who required radioiodine therapy. Nevertheless, a homogeneous patient collective was processed here, which could, for the first time, yield valuable results on the significance of stimulated and highly sensitive measured, unstimulated thyroglobulin measurements over a period of >10 years.

## Conclusion

U-hsTg measurement in patients with differentiated thyroid cancer within 24 months after completing radioiodine therapy showed higher predictive power than s-Tg measurement for RFS for a time period >10 years. If tumor marker Tg is undetectable at this time point, an s-Tg measurement appears to be dispensable (provided that disruptive TgAb interference can be ruled out in the individual case). However, one-third of patients with tumor recurrence had undetectable u-hsTg at u-hsTg1, indicating that periodic u-hsTg measurements still remain reasonable.

## Supplementary Material

Supplementary Material

## Declaration of interest

All other authors have nothing to declare. All disclosures were outside of the submitted work.

## Funding

This work did not receive any specific grant from any funding agency in the public, commercial or not-for-profit sector.

## Author contribution statement

Kim M. Pabst has received a Junior Clinician Scientist Stipend of the University Medicine Essen Clinician Scientist Academy (UMEA) sponsored by the faculty of medicine and Deutsche Forschungsgemeinschaft (DFG), travel fees from IPSEN Pharma and reports research funding from Bayer. Robert Seifert received research funding from the Boehringer Ingelheim Funds and the Else Kröner-Fresenius Stiftung. Manuel Weber is on the speakers bureau for Boston scientific. Wolfgang P. Fendler reports fees from SOFIE Bioscience (research funding), Janssen (consultant, speakers bureau), Calyx (consultant), Bayer (consultant, speakers bureau, research funding), Parexel (image review), Noveratis (speakers bureau), and Telix (speakers bureau). Timo Bartel has received travel fees from PARI GmbH. Ken Herrmann reports personal fees from Bayer, Sofie Biosciences, SIRTEX, Adacap, Curium, Endocyte, IPSEN, Siemens Healthineers, GE Healthcare, Amgen, Novartis, ymabs, Aktis, Oncology, Pharma15; non-financial support from ABX; grants and personal fees from BTG.
