# Supplementary material for: Predictive value of highly sensitive basal versus stimulated thyroglobulin measurement in long-term follow-up of thyroid cancer
Source: Endocr Connect. 2023 Jan 24;12(2):e220312. doi: 10.1530/EC-22-0312 (PMC9880903; doi:10.1530/EC-22-0312)
Supplement: Supplementary Material [file supplementary_material.pdf]

SUPPLEMENTS

Supplemental Table 1: List of patients with tumor recurrence – patient characteristics

Patient no.	Sex	Age (y)	Histology	Initial TNM	Radioiodine therapy dose (GBq)
1	m	34	PTC	T3(m) N1b M0	16.0
2	f	62	PTC	T4a N0 M0	9.0
3	m	56	FTC	T3 N0 M0	7.0
4	f	72	PTC	T1b N1b M0	9.0
5	m	63	FTC	T1b N0 M0	7.0
6	m	56	FTC	T3 N0 M0	9.0
7	m	73	FTC	T2 N0 M0	7.0
8	f	78	FTC	T2 N0 M0	7.0
9	f	29	FTC	T3 N0 M0	9.0
10	f	74	PTC	T3 N1b M0	3.0
11	m	57	PTC	T1b N0 M0	7.0
12	m	55	PTC	T3 N0 M0	9.0
13	f	48	FTC	T3 N0 M0	7.0
14	m	40	PTC	T3 N0 M0	9.0

GBq: Gigabecquerel, FTC: follicular thyroid carcinoma; PTC: papillary thyroid carcinoma.

Supplemental Table 2: Information about tumor recurrences

Patient no.	U-hsTg (ng/ml) at u-hsTg1	TSH (mU/l) at u-hsTg1	S-Tg (ng/ml) at s-Tg2	TSH (mU/l) at s-Tg2	U-hsTg (ng/ml) at u-hsTg3	TSH (mU/l) at u-hsTg3	Years until tumor recurrence*	Type of tumor recurrence
1	<0.09	0.11	1.82	135.74	3.9	0.01	10.0	SD / HC
2	<0.09	0.01	0.69	30.0	1.21	0.02	4.0	SD / HC
3	<0.09	0.02	0.21	111.81	2.2	0.01	3.0	SD / IC
4	0.09	0.4	0.56	53.8	0.38	0.01	0.5	SD / HC
5	0.11	0.01	0.89	35.6	19.0	0.09	4.0	SD / HC
6	0.28	0.2	2.0	66.23	61.0	0.01	2.0	SD / IC
7	<0.09	0.04	<0.09	41.79	2.5	0.48	6.0	SD / IC
8	0.29	0.03	1.3	81.0	0.96	0.43	3.0	SD /IC
9	0.14	0.1	1.98	60.05	2.0	0.01	4.0	SD / HC
10	0.38	0.01	3.6	110.0	39.0	0.04	11.0	BR / NC
11	<0.09	0.02	1.7	45.0	1.03	0.15	8.0	SD / HC
12	0.64	0.05	9.0	66.0	12.0	0.03	2.0	SD / IC
13	0.64	0.01	1.8	44.0	1911.0	0.02	5.0	SD / IC
14	0.44	0.01	44.0	38.0	0.4	0.01	0.0	SD / HC

***After stimulated thyroglobulin measurement as part of radioiodine diagnostic.

U-hsTg: unstimulated, highly sensitive measured thyroglobulin, s-Tg: stimulated thyroglobulin, u-hsTg1: u-hsTg measurement 6±3 months before s-Tg, s-Tg2: s-Tg

measurement ≤24 months after completion of primary therapy, u-hsTg3: last u-hsTg before diagnosis of tumor recurrence, BR: biochemical tumor recurrence; HC: histologically confirmed; IC: confirmed by imaging; NC: not confirmed; SD: structural disease.

Supplemental Table 3: Localization of tumor recurrences

**Localization of tumor recurrence**	**N (%)**
**Local**	**1 (7.1)**
**Lymph nodes**	**6 (42.9)**
Cervical	5 (35.7)
Mediastinal/hilar	1 (7.1)
**Distant metastases**	**8 (57.1)**
Pulmonary	5 (35.7)
Hepatic	1 (7.1)
Osseous	2 (14.3)

Localization of tumor recurrences of the studied patient population with differentiated thyroid carcinoma subdivided according to local recurrence, lymph node metastases, and distant metastases.
